# BMD Loci Contribute to Ethnic and Developmental Differences in Skeletal Fragility across Populations: Assessment of Evolutionary Selection Pressures

**DOI:** 10.1093/molbev/msv170

**Published:** 2015-07-29

**Authors:** Carolina Medina-Gómez, Alessandra Chesi, Denise H.M. Heppe, Babette S. Zemel, Jia-Lian Yin, Heidi J. Kalkwarf, Albert Hofman, Joan M. Lappe, Andrea Kelly, Manfred Kayser, Sharon E. Oberfield, Vicente Gilsanz, André G. Uitterlinden, John A. Shepherd, Vincent W.V. Jaddoe, Struan F.A. Grant, Oscar Lao, Fernando Rivadeneira

**Affiliations:** ^1^Department of Internal Medicine, Erasmus University Medical Center, Rotterdam, The Netherlands; ^2^The Generation R Study Group, Erasmus University Medical Center, Rotterdam, The Netherlands; ^3^Department of Epidemiology, Erasmus University Medical Center, Rotterdam, The Netherlands; ^4^Division of Human Genetics, Children's Hospital of Philadelphia, Philadelphia, PA; ^5^Department of Pediatrics, Erasmus University Medical Center, Rotterdam, The Netherlands; ^6^Division of GI, Hepatology, and Nutrition, Children's Hospital of Philadelphia, Philadelphia, PA; ^7^Department of Pediatrics, Perelman School of Medicine, University of Pennsylvania; ^8^Division of General and Community Pediatrics, Cincinnati Children’s Hospital Medical Center, Cincinnati, OH; ^9^Division of Endocrinology, Creighton University, Omaha, NE; ^10^Department of Genetic Identification, Erasmus University Medical Center, Rotterdam, The Netherlands; ^11^Division of Pediatric Endocrinology, Diabetes, and Metabolism, Department of Pediatrics, Columbia University Medical Center, New York, NY; ^12^Department of Radiology, Children’s Hospital Los Angeles, Los Angeles, CA; ^13^Department of Radiology and Biomedical Imaging, University of California, San Francisco

**Keywords:** genome-wide association studies, bone mineral density, polygenic, adaptation, ethnic differences, selective pressures

## Abstract

Bone mineral density (BMD) is a highly heritable trait used both for the diagnosis of osteoporosis in adults and to assess bone health in children. Ethnic differences in BMD have been documented, with markedly higher levels in individuals of African descent, which partially explain disparity in osteoporosis risk across populations. To date, 63 independent genetic variants have been associated with BMD in adults of Northern-European ancestry. Here, we demonstrate that at least 61 of these variants are predictive of BMD early in life by studying their compound effect within two multiethnic pediatric cohorts. Furthermore, we show that within these cohorts and across populations worldwide the frequency of those alleles associated with increased BMD is systematically elevated in individuals of Sub-Saharan African ancestry. The amount of differentiation in the BMD genetic scores among Sub-Saharan and non-Sub-Saharan populations together with neutrality tests, suggest that these allelic differences are compatible with the hypothesis of selective pressures acting on the genetic determinants of BMD. These findings constitute an explorative contribution to the role of selection on ethnic BMD differences and likely a new example of polygenic adaptation acting on a human trait.

## Introduction

Enormous progress in mapping complex traits has been achieved in the last decade with thousands of loci identified by the implementation of genome-wide association studies (GWAS) (Visscher et al. 2012). While individually, variants at these loci each make modest contributions, collectively, the effect of large sets of variants increase significantly the amount of explained trait variance. These discoveries have also enabled studying how selective pressures influence the genetic architecture of complex traits across populations. For some phenotypes such as skin pigmentation (McEvoy et al. 2006), type 2 diabetes (Chen et al. 2012), height (Turchin et al. 2012), biliary liver cirrhosis, and ulcerative colitis (Corona et al. 2013), there is increasing evidence that the genetic basis of trait differences across human populations has been shaped by evolutionary selective pressures (Berg and Coop 2014).

Just as in most other medical areas, the field of genetics of osteoporosis has also been revolutionized by the advent of the GWAS approach, with up to 63 independent genetic variants identified as robustly associated with bone mineral density (BMD) in adults of Northern-European ancestry (Estrada et al. 2012). BMD measured by dual-energy X-ray absorptiometry (DXA) is a highly heritable trait used to diagnose osteoporosis and assess the risk of fracture (Cummings et al. 1993). BMD levels are determined by the processes of bone accrual (throughout childhood until young adulthood) and bone loss (from peak bone mass acquisition to senescence). Ethnic differences in BMD are well documented and partially explain differences in osteoporosis and fracture risk across populations. Individuals of Sub-Saharan African ancestry tend to have higher BMD levels and lower fracture risk as compared with other populations (Finkelstein et al. 2002; Marshall et al. 2008).

It is likely that the skeletal system as a whole (and its derived bone strength) has been subjected to selective pressures throughout human evolution (Nowlan et al. 2011). This includes changes brought about through human history ranging from early nomadism to the domestication of animals, crops and even more recently, a tendency to ever increasing levels of sedentariness (Ryan and Shaw 2015). In order to understand how selective pressures have shaped bone strength variation across human populations we 1) studied the collective effect of BMD-associated variants within two independent multiethnic pediatric cohorts; 2) examined the allele frequency distribution of these variants across diverse ethnic populations using two different catalogues of genetic variation; and 3) assessed the presence of selective pressures by testing for deviation from genetic drift.

## Results

### Total Body BMD Differences across Ethnic Ancestries

**Fig. 1.**
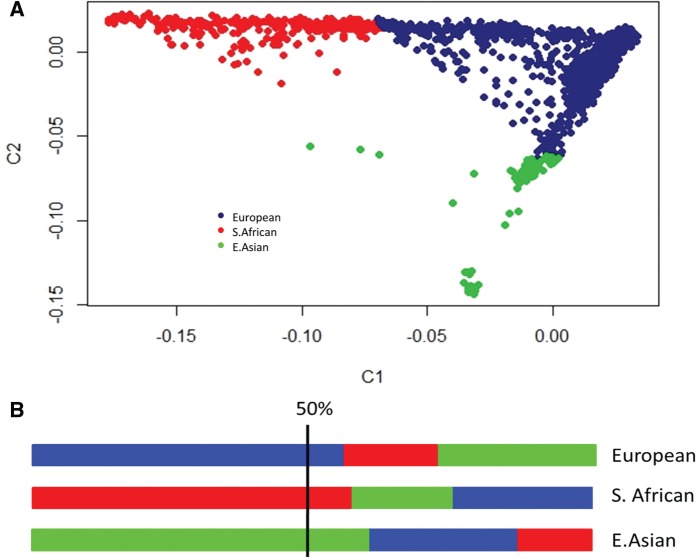
Genetic substructure of the Generation R Study. (*A*) Two-dimensional plots from MDS analyses of the Generation R Study based on the first two genomic principal components. (*B*) Clustering rule based on ancestral proportions. Children were assigned to one of the three main genetic ancestry groups based on their highest fraction of estimated ancestry (i.e., >0.50) proportion: European (blue), Sub-Saharan African (red), and East Asian (green). Individuals with no ancestry proportion greater than 0.50 were excluded.

To characterize pediatric ethnic differences in BMD, we selected a sample of 3,994 children (mean age of 6 years) of multiethnic background taking part in the Generation R Study (Jaddoe et al. 2012), who had GWAS data and DXA measurements. First, we determined genetic population substructure from genotyped data, children were assigned to one of the three main genetic ancestry groups (fig. 1): Sub-Saharan African (*n* = 336), European (*n* = 3,499), and East Asian (*n* = 159) descent. Table 1 summarizes the characteristics of the Generation R participants under study. Children of European and East Asian ancestry had respectively, 4% (0.552 g/cm^2^, *P* < 2 × 10^−^^16^) and 2.8% (0.559 g/cm^2^, *P* = 7 × 10^−^^6^) lower BMD than children who clustered in the Sub-Saharan African group (0.575 g/cm^2^), after controlling for age, sex, height, fat, and lean mass. These differences remained statistically significant after correction for lifestyle factors affecting mothers and children. Furthermore, we observed that, in European children, the proportion of genetic Sub-Saharan African ancestry in their genomes (range 0.00–0.49) was positively associated with BMD levels, representing a BMD increase of 0.0003 g/cm^2^ per increase in genomic Sub-Saharan African component (*β* = 0.72, *P* = 1.87 × 10^−^^5^). Thus, the BMD difference between an individual with 0% Sub-Saharan African ancestry and one with 49.99% Sub-Saharan African ancestry in their genomes will be equivalent to 0.016 g/cm^2^, suggesting that genetic factors play a role in the observed ethnic BMD differences.

**Table 1.** Participant Characteristics in the Generation R Study and BMD Childhood Study by Defined Genetic Ancestry.

	Generation R Study (The Netherlands)								BMD Childhood Study (United States)							
	S. African		European		East Asian		All		S. African		European		East Asian		All	
	*n *= 336		*n *= 3,499		*n *= 159		*n *= 3,994		*n *= 315		*n *= 1,277		*n *= 78		*n *= 1,670	
Age, years	6.41	0.68	6.15	0.46	6.20	0.42	6.18	0.50	11.45	4.46	11.4	4.41	12.27	4.68	11.46	4.43
Women (*n*, %)	171	50.9	1,747	50.1	72	54.7	2,001	50.1	165	52.4	666	52.2	35	44.9	866	51.9
Height, m	1.219	0.07	1.195	0.06	1.177	0.06	1.196	0.06	1.452	0.21	1.446	0.22	1.455	0.21	1.447	0.22
Weight, kg	24.48	4.99	22.94	3.87	21.64	4.50	23.06	4.05	43.31	18.56	42.24	19.02	43.6	18.28	42.5	18.9
Fat mass, kg	6.397	2.90	5.787	2.21	5.947	2.71	5.844	2.30	9.88	6.00	10.11	5.87	10.69	6.20	10.10	5.90
Lean mass, kg	17.67	2.80	16.33	2.20	14.92	2.38	16.39	2.28	32.05	13.90	31.06	14.40	30.99	13.50	31.24	14.30
BMD, g/cm^2^	0.595	0.06	0.551	0.046	0.541	0.055	0.555	0.05	0.828	0.198	0.775	0.192	0.798	0.199	0.786	0.195

Note.—Data are means and SD (except for women number and percentage). S.African, Sub-Saharan African.

We confirmed the existence of these ethnic differences in BMD in an independent multi-ethnic set of 1,670 US children from the Bone Mineral Density in Childhood Study (BMDCS), using the same ethnic definition and analytical approach. Characteristics of the BMDCS participants under study are presented in table 1. Consistent with our findings, after adjustments children of European (*n* = 1,277) and East Asian (*n* = 78) ancestry had 4.7% (0.778 g/cm^2^, *P* = 0.0002) and 2.5% (0.794 g/cm^2^, *P* < 2 × 10^−^^16^) lower BMD levels than children of Sub-Saharan African ancestry (*n* = 315) with mean BMD 0.816 g/cm^2^, respectively.

### BMD Genetic Score Association with Pediatric BMD

We constructed a genetic score—composed of BMD-increasing alleles—for each child using single nucleotide polymorphisms (SNPs) known to be associated with BMD in adults of Northern-European ancestry (Estrada et al. 2012) (supplementary table S1, Supplementary Material online). In the Generation R Study, the calculated BMD genetic score (BMD-GS) explained 4.5% of the BMD variance in all the sampled children (*P* < 2 × 10^−^^16^). Additional correction by ten genomic principal components, addressing potential residual population stratification, indicated that up to 2.6% of BMD variation is explained by the BMD-GS (*P* < 2 × 10^−^^16^). In children from the BMDCS, the BMD-GS explained 5.7% and 2.2% of the BMD variance, before and after correction by ten principal components (supplementary table S2, Supplementary Material online). The BMD-GS was positively associated with BMD in children of predominantly European and African descent of both studies, albeit the latter reached nominal significance only in the BMDCS cohort. In children of predominantly East Asian ancestry (with the lowest sample size) the effect of the score on BMD did not reach statistical significance in either study. Joint analysis of the Generation R and BMDCS resulted in significant associations of BMD with the BMD-GS in children of European (*β* = 4.43, *P* < 2 × 10^−^^16^) and Sub-Saharan African (*β* = 2.41, *P* = 0.016) background, but not in those of East Asian (*β* = 1.18, *P* = 0.472) descent.

We then examined BMD levels across quintiles of the calculated BMD-GS in the two pediatric cohorts. There was a positive correlation between the score and the adjusted BMD in both studies. In the Generation R Study, as compared with children in the middle quintile (55.8% of the population with the mean BMD; *n* = 2,232), children in the highest quintile (2.2% of the population; *n* = 88) had 0.72 standard deviations (SDs) higher BMD (*P* < 1 × 10^−^^6^), whereas those in the lowest quintile (1.3% of the population; *n* = 53) had 0.53 SDs (*P* = 4 × 10^−^^6^) lower BMD. Similar results were obtained in the BMDCS study where children in the highest quintile (1.1% of the population; *n* = 19) had 0.42 SDs higher BMD (*P* = 0.06) and those in the lowest quintile (2.9% of the population; *n* = 49) 0.55 SDs lower BMD (*P* = 0.001) as compared with the children in the middle quintile (51.3% of the population, *n* = 860).

**Fig. 2.**
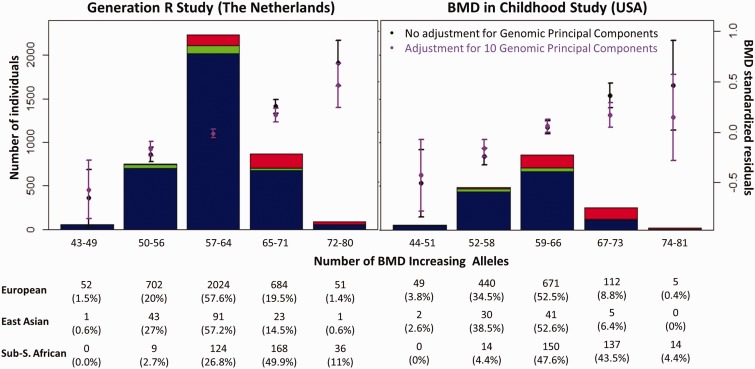
Distribution of the BMD-increasing allele score by genetic ancestry in the Generation R Study and BMD in Childhood Study. The increasing BMD-GS has been divided in five category bins. Colors in the stacked bars represent ethnic background: European (blue), East Asian (green), and Sub-Saharan African (red). Black dots represent the mean BMD adjusted for age, gender, height, fat, and lean mass. Magenta dots represent the mean BMD per bin, adjusted for all former variables plus the first ten principal components.

As depicted in figure 2, the overrepresentation of children of Sub-Saharan African descent across the higher quintiles of the BMD-GS was highly significant in both studies (Generation R: *P* = 2.7 × 10^−^^71^ and BMDCS, *P* = 2.3 × 10^−^^70^). In the Generation R Study 60.7% of children of Sub-Saharan African ancestry are found in the two highest GS quintiles, as compared with children of European (20.8%) and East Asian (15.1%) ancestry. For the BMDCS, 47.9% of children of Sub-Saharan African ancestry are found in the two highest GS quintiles, as compared with children of European (9.2 %) and East Asian (6.4%) ancestry. In both pediatric cohorts the mean BMD-GS values were highest in the Sub-Saharan African group (Generation R: 0.537, BMDCS: 0.542), while remaining similar in the European (Generation R: 0.495, BMDCS: 0.493) and East Asian (Generation R: 0.487, BMDCS: 0.491) groups.

Computing BMD-increasing allele scores using additional sets of markers associated with BMD at lower levels of significance showed that the greatest difference in mean GS values between subjects of African and European background was observed with the original BMD-GS including 61 markers (supplementary fig. S1, Supplementary Material online). The addition of more (less significantly associated) SNPs to the score decreased the mean allele score difference observed between the two ethnic groups and did not improve significantly the BMD explained variance.

### Worldwide Geographical Distribution of the BMD-Increasing Alleles

We then sought to understand the observed ethnic mean differences in BMD-GS by decomposing it and considering each of the BMD-increasing alleles independently. In the Generation R Study, where the sample size allows more accurate statistical inferences, we found that in children of Sub-Saharan African ancestry 23 of these alleles had ≥10% higher frequency as compared with only 12 in Europeans (*P* = 0.04). When examining HapMap data (International HapMap Consortium 2003), similar allele frequency differences were observed between the CEU (Utah residents of European Ancestry) and YRI (Yoruba people from Ibadan, Nigeria) populations, independent of the CEU SNP minor allele frequency (MAF) (supplementary table S1, Supplementary Material online).

**Fig. 3.**
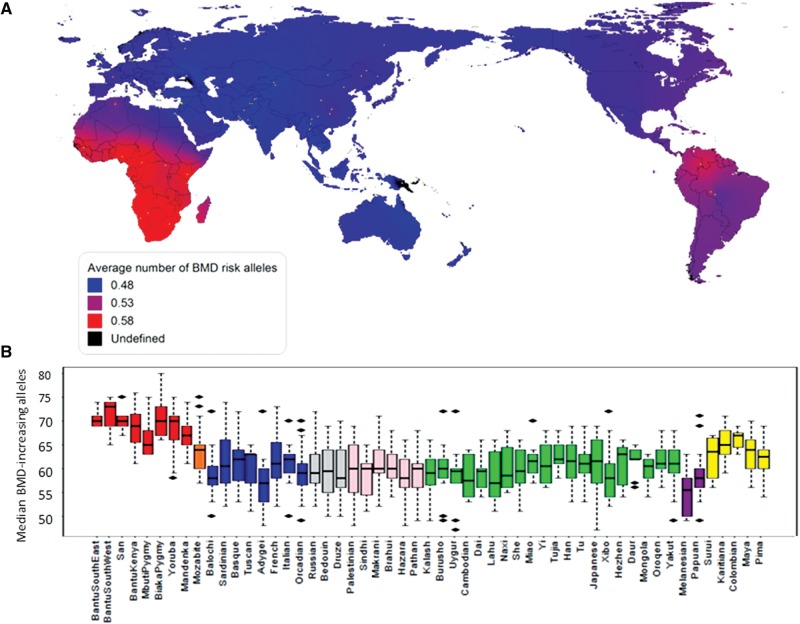
Worldwide geospatial distribution of BMD-increasing alleles. (*A*) The density map of the combined frequency of the BMD-GS was generated by inverse to distance interpolation using the observed values across the 53 HGDP populations. (*B*) Number of BMD-increasing alleles per individual in the 53 populations of the HGDP. Populations are ordered by geographic groups: Sub-Saharan Africa (red), North-Africa (orange), Europe (blue), Middle East (gray), Central Asia (pink), East Asia (green), Oceania (purple), and Native Americans (yellow).

In order to broaden the scope of our findings, we analyzed the spatial distribution of the BMD-GS SNPs worldwide, using the populations from the Centre D'Etude du Polymorphism Humaine-Human Genome Diversity Project (CEPH-HGDP) panel (Li et al. 2008) (supplementary table S3, Supplementary Material online). As shown on figure 3, examining the BMD-GS across all individuals from the different populations, we confirmed that Sub-Saharan African populations had the highest number of BMD-increasing alleles, together with other two Latin-American populations located in the tropics. Likewise, the populations out of Africa in general had lower BMD-GS scores.

### Signatures of Natural Selection in the BMD-GS SNPs and Associated Regions

We followed sixth different lines of evidence to discard that genetic drift was explaining the observed spatial distribution of the mean BMD-GS (fig. 3). First, we observed that the BMD-increasing alleles were enriched for ancestral alleles (73% actually constitute ancestral alleles; *P* = 1.8 × 10^−^^4^). Second, taking advantage of the geographical coverage of the HGDP, we quantified the differentiation between continents, where for each HGDP individual we generated 100,000 permuted scores by randomizing the coded effect allele in each of the BMD-GS variants. The BMD-GS differentiation between Sub-Saharan African and non-Sub-Saharan African populations was higher for the original BMD-GS (*P* = 0.025) than for the different sets of sampled scores. Third, in line with the previous test, we evaluated the population-specific departure of the BMD-GS score from its expected genomic value and determined that the proportion of variance attributable to the categorization into Sub-Saharan African and non-Sub-Saharan African populations, was significantly higher (*P* = 0.011) for the original BMD-GS than that observed in 100,000 GS sets of 61 SNPs randomly sampled from the genome (after matching for factors related to GWAS discovery bias). These results provide additional statistical support to the contention that the observed differentiation of Sub-Saharan African populations, as shown by the multidimensional scaling (MDS) and ADMIXTURE analyses using only the markers of the BMD-GS (supplementary fig. S2, Supplementary Material online), is not due to genetic drift. Fourth, we estimated the Q_X_ statistic (Berg and Coop 2014), which analyzes extreme patterns of genetic differentiation among populations, which was not significant (Qx = 46.67, *P* = 0.68). Fifth, to assess if selective pressures acting on the score explained these results, we scrutinized the YRI, CHB (Han Chinese individuals from Beijing, China), and CEU populations and found no signals of strong selective sweeps acting on the loci harboring the BMD-GS SNPs (supplementary table S4, Supplementary Material online). Nonetheless, we did find evidence for departure from neutrality in CEU and CHB (Hierarchical Bayesian binomial modeling, and supplementary table S5, Supplementary Material online); resulting in a possible excess of intermediate-frequency alleles in the BMD-GS. Sixth, we applied tests for differences in mean MAF and determined that as compared with SNP sets randomly ascertained from the GWAS catalogue, the SNPs of the BMD-GS have significantly higher (*P* = 8.3 × 10^−^^3^) mean MAF in European (MAF = 0.30 in CEU), but not in East Asian (MAF = 0.26; *P* = 0.13 in JPT [Japanese population from the Tokyo area, Japan] + CHB) or African (MAF = 0.21; *P* = 0.83 in YRI) populations. Altogether, five out of sixth different lines of evidence argue against genetic drift explaining the observed ethnic differences in mean BMD-GS values.

## Discussion

In this study, we established that ethnic differences in BMD are already present early in life and are partially explained by variation in BMD-associated loci. Furthermore, we demonstrate in two independent pediatric multiethnic cohorts that those alleles positively associated with BMD are systematically elevated in populations of Sub-Saharan African ancestry. The same pattern was also observed in a broader framework of catalogues of human genetic variation. Five out of six tests indicate deviation from neutrality and are suggestive of polygenic adaptation acting on the loci harboring the variants of the score. Our results confirm that the Sub-Saharan African versus non-Sub-Saharan African distribution of the BMD increasing alleles worldwide cannot be simply explained by demographic factors or genetic drift.

We assessed the existence of ethnic differences in BMD levels first within the Generation R Study. In contrast to ethnic comparisons conducted across different geographical areas (Finkelstein et al. 2002; Zemel et al. 2011; Nam et al. 2013), these research subjects were relatively standardized as they were all residents of the city of Rotterdam, The Netherlands; measured using the same DXA device; and within a similar age range. This study design reduces the impact of intermachine variance, as well as local environmental and lifestyle exposures influencing BMD variation such as major differences in access to medical care, sunlight exposure, diet, or physical activity. Children of Sub-Saharan African ancestry showed higher BMD in this set even after correction for lifestyle factors affecting both mothers and children. Nevertheless, because each child might have a varying degree of each of the three ancestral populations in their genome (fig. 1), actual ethnic BMD differences might be even higher than reported here when more stringent ethnic definitions are used. These differences found in the Generation R study and replicated in the BMDCS, together with the positive association of Sub-Saharan African genomic ancestry proportion and BMD, suggest that genetic factors play a role in the observed phenotypic differences in BMD.

The fact that the BMD-GS was significantly associated with BMD in children from both the Generation R and BMDCS (fig. 2), implicates developmental effects of the studied variants acting on the bone accrual process. The systematic higher frequency of the BMD increasing-alleles in children of Sub-Saharan ancestry mirrored their higher BMD, advocating that ethnic differences in BMD are to a given extent genetic in origin and not likely due to population stratification (enduring correction for genomic principal components).

Our results indicate that the BMD-GS is more predictive in subjects of European descent (where variants were discovered) than in other ethnic groups. Nevertheless, differences in the effect of the score across ethnic populations in our pediatric studies could simply reflect greater uncertainty in estimating effect sizes of the BMD-SNPs for these populations given their small sample size. Despite not finding an association in children of East Asian background, previous studies in East Asian populations have replicated associations with BMD for several variants of the genetic score (Styrkarsdottir et al. 2010; Deng et al. 2013; Ham and Roh 2014). In fact, high trans ethnic replicability has been described between these populations for other complex traits (Marigorta and Navarro 2013). Additionally, ethnic differences in the degree of association of the BMD-GS can also reflect population differences in the relationship between tagging and true underlying genetic variants (Marigorta and Navarro 2013). This is especially evident in African populations where blocks of linkage disequilibrium (LD) are the shortest and associations with phenotypes can result in dilution of effects (Carlson et al. 2013). Therefore, the true directional population differentiation at causal SNPs could be even larger than the directional population differentiation observed in tagging SNPs, likely comprising a large fraction of the variants in the GS we employed. Further, other variants more frequent in African or Asian populations may contain additional set of alleles contributing to BMD variation in those groups; this contention is also supported by the significant association of BMD with the fraction of Sub-Saharan African ancestry in Europeans, which remained significant independently of the BMD-GS.

In the genetic factors of osteoporosis (GEFOS) BMD meta-analysis, a weighted version of the BMD-GS explained 5.8% of the femoral neck BMD variance in a cohort of unrelated postmenopausal Danish women (Estrada et al. 2012). The BMD-GS explained relatively lower variance in our two pediatric multiethnic cohorts (Generation R: 2.6%, BMDCS: 2.2%), likely as consequence of the different weighting scheme, age range (Medina-Gomez et al. 2012), skeletal site specificity (Kemp et al. 2014), or ethnic context. The use of a weighted scores could increase predictive ability but also introduce possible bias due to the above mentioned differences in study setting, as well as residual population stratification intrinsic in GWAS studies (Turchin et al. 2012).

By interrogating publicly available databases we could establish that the results observed in the two pediatric cohorts were paralleled in a worldwide scenario (fig. 3). Frequency differences in the Generation R Study reflected the differences observed in the HapMap YRI and CEU populations (International HapMap Consortium 2003). The magnitude of these differences was such, that by employing only the BMD-GS SNPs we could unequivocally separate Sub-Saharan African individuals from all others in the populations from the HGDP-CEPH panel (supplementary fig. S2, Supplementary Material online). This geographic pattern is in line with the geographical disparities in fracture risk worldwide and parallel a potential association with latitude (Cummings and Melton 2002; Cauley 2011; Kanis et al. 2012; Nilson et al. 2014). However, we did not find any clear BMD-GS south to north gradient among the HGDP-CEPH populations. Yet, an association with latitude may be attenuated as the populations part of the HGDP-CEPH panel are unevenly distributed (Xing et al. 2010). Likewise, careful consideration is needed when drawing conclusions about findings in African populations, as Africa is a continent characterized by profound genetic diversity and home to a vast array of heterogeneous lifestyles (Tishkoff et al. 2009). Given the limited representation of African populations in the HGDP-CEPH and HapMap data sets, the association between the BMD-GS and Sub-Saharan African ancestry is not to be directly extrapolated across the whole African continent. Nevertheless, our findings suggest that a fraction of the population differences in BMD levels are genetic in origin, without disregarding the large environmental influences contributing to trait variation and ethnic differences.

The overrepresentation of ancestral alleles in the BMD-GS, indicates that phenotypic states related to increased BMD, such as bone robustness (Nowlan et al. 2011), represent the ancestral state in humans and show an amount of phenotypic differentiation that cannot be uniquely explained by demographic factors. A similar result was observed by Wu and Zhang (2010) when analyzing genes that had been associated with the skeletal system in humans. The authors concluded that such excess of derived variants in populations from European and East Asian ancestry was suggestive of selective pressures out of the African continent, although this could also be consequence of a relaxed constraint. We observed that the BMD-GS differentiation between Sub-Saharan African and non-Sub-Saharan African populations was significantly larger than expected under neutrality for both the randomization test of allele effects and when compared with the rest of the genome. In fact, departure of the BMD-GS from neutrality was supported by five of six different types of tests, including: 1) detection of enrichment of ancestral alleles in the set of BMD increasing alleles in the BMD-GS; 2) randomization of the BMD increasing allele and quantification of the differentiation between continents; 3) population departure of the expected genomic value of BMD-GS; 4) detection of departure of neutrality in sequence-based tests; and 5) presence of an excess of intermediate allele frequencies. We acknowledge that the statistical evidence derived from these five tests was marginal. However, such magnitude of statistical significance is in agreement with those reported for other complex phenotypes (cf. Corona et al. 2013; Perry et al. 2014). Only on the 6) Qx index test (Berg and Coop 2014), we did not find any evidence for departure from neutrality. This discrepancy could be due to the wide range of hypotheses about differentiation considered by the Qx, which models all different population levels. In contrast, the other tests (i.e., analysis of variance [ANOVA]) were motivated by the empirical observation that the BMD-GS is increased in Sub-Saharan populations and applied specifically to detect differences between two groups (Sub-Saharan vs. non-Sub-Saharan African populations). Our results, (supplementary table S4, Supplementary Material online) as expected under polygenic adaptation, showed no enrichment for strong selective sweeps in the BMD-associated loci but rather displayed small allele frequency shifts between populations (Pritchard et al. 2010). One plausible explanation is that methods for detecting strong selective sweeps are under powered in regions containing a relative excess of intermediate-frequency alleles compared with the rest of the genome. We observed that in the European populations the BMD-GS SNPs have a systematic excess of intermediate-frequency alleles as compared with that observed in other SNPs reported in the GWAS catalogue (Welter et al. 2014). This footprint of intermediate-frequency alleles in the BMD-GS SNPs can be produced by the presence of selective pressures increasing the genetic variability, such as balancing or directional selection on standing variation (Przeworski et al. 2005) or by SNP ascertainment bias on the GWAS discoveries (Casto and Feldman 2011). Nevertheless, since our sampling SNPs sets were drawn from the GWAS catalogue, and controlled for multiple putative confounder factors, ascertainment bias is an unlikely explanation.

The skeletal system as a whole and its derived bone strength are likely to have been subject to natural selection during human evolution (Nowlan et al. 2011). Multiple factors may have been responsible for selective pressures such as: long distance running and trekking (indispensable for hunting and scavenging), the establishment of a more sedentary lifestyle (with the advent of agriculture), which would have favored skeletal gracility (Nowlan et al. 2011; Ryan and Shaw 2015); adaptation to less sunlight at more distant latitudes from the equator (i.e., skin color and vitamin D metabolism) or differences in milk/calcium intake (i.e., lactase persistence), among several others. Therefore, it is conceivable that evolution would favor an optimal balance between bone thickness and bone strength within the context of the structural and metabolic functions that the skeleton must serve. However, the ultimate reason for the presence of this genetic signature “out of Africa” remains to be elucidated. Understanding of the evolutionary and genetic mechanisms shaping BMD variation and peak bone mass acquisition across populations will facilitate pinpointing critical factors underlying ethnic differences in the risk of osteoporosis later in life.

In summary, we provide evidence that disparities in global patterns of BMD-increasing allele frequencies contribute to disparities in BMD levels across different ethnicities, effects which are already present at early ages, postulating a critical role of these variants in the developmental process of peak bone mass accrual. We also show that these allelic differences are compatible with the hypothesis of selective pressures acting on the genetic determinants of BMD. These findings constitute an explorative contribution to the role of selection on ethnic BMD differences and likely a new example of polygenic adaptation acting on a human trait.

## Materials and Methods

### Study Populations

#### The Generation R Study

It is a population-based prospective cohort study from foetal life onwards in Rotterdam, the Netherlands (Jaddoe et al. 2012). Data from a total of 3,994 children with blood collected at birth, subsequently genotyped, and valid DXA scans at 6 years of age were included in these analyses. Participants underwent DXA measurements with a GE-Lunar iDXA device (GE Healthcare Lunar, Madison, WI) following standard manufacturer protocols. DNA samples were genotyped either on the Illumina HumanHap 610 or Illumina HumanHap 660 chip. Duplicated samples or with excess of heterozygocity and gender mismatches were excluded from the data set. SNPs with a MAF < 1%, call rate<98% or out of Hardy–Weinberg equilibrium (*P* < 10^−^^6^) were removed from further analyses. Imputations to the combined HapMap Phase II Build 36 Release 22 panel (International HapMap Consortium 2003), composed of dense genotypes from four populations: CEU, YRI, and CHB + JPT, were performed following a two-step procedure as implemented in the MACH/minimac suit.

#### Bone Mineral Density in Childhood Study

BMDCS is a longitudinal multicenter study in the U.S. designed with the goal of obtaining standard pediatric reference data for BMD assessed using DXA (Kalkwarf et al. 2007; Zemel et al. 2011). In total, 2,521 boys and girls aged 5–20 years old were recruited between 2002 and 2007 and DXA measurements were obtained annually at five clinical centers in the United States for up to seven measurements. Another 507 children of European ancestry aged 5–18 year old were subsequently enrolled for a one-time visit in two of the five centers. Enrollment criteria were established to identify research subjects with normal development, including healthy bones. DXA scans were obtained using Hologic, Inc. (Bedford, MA) bone densitometers. The baseline measurements for 1,670 individuals were used for this analysis. All samples were genotyped on the HumanOmniExpressExome-8v1 chip. Samples with gender discrepancy, low genotype quality, sample replicates, and siblings were excluded from the analysis. SNPs with MAF less than 0.5% and call rate of less than 95% were removed. Imputation was performed following a two-step procedure; haplotype phasing was carried out using ShapeIT while imputation to the combined HapMap Phase II Build 36 Release 22 panel was performed with Imputev2.

#### Centre D'Etude du Polymorphism Humaine-Human Genome Diversity Project Panel

HGDP-CEPH is a collection of DNA samples from lymphoblastoid cell lines representing 1,064 individuals sampled from 53 populations throughout the world (Li et al. 2008). We downloaded Illumina *650Y* data for 1,043 samples from the HGDP-CEPH (http://www.hagsc.org/hgdp/files.html, last accessed February 2, 2014). After excluding duplicates, ethnical outliers, first or second degree relatives (Rosenberg 2006) and individuals with more than 2% missing SNPs, we selected a panel comprising 940 individuals (supplementary table S3, Supplementary Material online). SNPs were removed if the MAF was less than 1%, call rate less than 98% or if they were out of Hardy–Weinberg equilibrium (*P* < 10^−^^6^). All samples were imputed to the combined HapMap Phase II Build 36 Release 22 panel. Imputations were performed following a two-step procedure as proposed by the MACH/minimac suit.

### Individual BMD Estimation

Total body BMD was measured in the two population-based studies (Generation R Study and the BMDCS) using DXA scans. In both studies measurements were conducted by well-trained research assistants and daily quality control assurance was performed. Prior to the scan procedure, participants were asked to take off their shoes, heavy clothes, and metal accessories. As recommended by the International Society for Clinical Densitometry (Lewiecki et al. 2008), total body less head BMD was the measurement used in the analysis, as both studies are embedded in pediatric populations.

#### BMD-Associated SNPs

For this study, we used 61 out of the 63 autosomal SNPs reported as genome wide associated with either Femoral Neck or Lumbar Spine BMD (*P* ≤ 5 × 10^−^^8^) in the large scale GWAS meta-analyses of BMD from the GEFOS (Estrada et al. 2012) successfully imputed in the different study populations. Description of these SNPs can be found in the supplementary table S1, Supplementary Material online. Comparison of BMD-increasing allele frequency was completed based on the Generation R children of Sub Saharan African and European ancestry and on the YRI and CEU HapMap project (International HapMap Consortium 2005) populations, in dbSNP (Sherry et al. 2001).

### Estimation of Genomic Principal Components and Genetic Ancestry Determination

Ancestry determination was performed in each of the studies, Generation R and BMDCS, separately but following the same protocol. Autosomal genotyped SNPs of all samples were pruned, so that no pair of SNPs within a window of 200 markers were in LD (*r*^2 ^= 0.05). Based on these approximately 35,000 SNPs, we described the genetic ancestry of the whole data set by means of classical MDS in PLINK, generating 10 genomic principal components. We then performed estimation of the genetic ancestry components of each individual on basis of the maximum likelihood using ADMIXTURE software (Alexander et al. 2009). This program models the probability of observed genotypes using ancestry proportions and ancestral population allele frequencies. The clustering method was set to group individuals in three ancestral populations (*K* = 3), corresponding to the expected main Sub-Saharan African, European and East Asian ancestry components. Children were assigned to one of the three ancestry groups, labeled after the HapMap Phase II populations, based on their highest fraction of estimated ancestry (i.e., >0.50) proportions. In case none of the three ancestral populations reached this proportion, the participant was excluded from further analyses. Ancestry determination was also calculated for the HGDP individuals based exclusively in the 61 BMD-associated variants and assuming *K* = 3 ancestral proportions. Furthermore, we computed genomic principal components using the MDS routine and clustered the individuals with the R package Mclust (http://cran.r-project.org/web/packages/mclust/index.html, last accessed April 10, 2014) This algorithm assigns individuals to clusters by fitting multivariate normal distributions using the coordinates of the proposed dimensions and put forward the best clustering based on the Bayesian Information Criterion.

### BMD-GS Calculation

Scores were obtained for each individual as an unweighted sum across the 61 BMD-associated SNPs of the number of putative increasing alleles (0, 1, or 2) using the profile scoring routine in PLINK. BMD-GS calculations were performed for each population independently based on best guess genotypes obtained using GCTA software (Yang et al. 2011) after imputation and quality control of the imputed data set. Extensions of the score to include SNPs associated at the meta-analysis level with BMD at different significance thresholds were also generated. We used the PLINK clumping function, following a strategy described elsewhere (Turchin et al. 2012). Briefly, we combined publicly available results of the stage 1 lumbar spine and femoral neck BMD meta-analyses (Estrada et al. 2012), data publicly available (http://www.gefos.org/?q=content/data-release, last accessed March 20, 2015). We assigned to each SNP the lowest *P* value reached genome-wide in any of the two association analyses by skeletal site. Then we clumped the data set with an *r*^2^ ≥ 0.1 using as reference the most BMD-associated SNPs and pruned remaining SNPs within 0.5 Mb of each other. Using different thresholds of significance (5 × 10^−^^8^, 5 × 10^−^^7^, 5 × 10^−^^6^, 5 × 10^−^^5^, 5 × 10^−^^4^, 5 × 10^−^^3^, 0.05 and 0.1) we generated eight different scores. Each cumulative threshold set contains the SNPs included in sets defined by previous thresholds (e.g., the set with threshold 5 × 10^−^^6^ includes all the SNPs with a *P* value smaller than 5 × 10^−^^6^, comprising those at *P* value thresholds of 5 × 10^−^^8^, 5 × 10^−^^7^, etc). For each score we calculated both the BMD explained variance and the mean difference of BMD increasing alleles between children of Sub-Saharan and those of European ancestry. Some differences between our score and the one including only GWS SNPs apply. The GEFOS meta-analysis involved a two-stage meta-analysis. In the first stage of the meta-analysis (discovery), only GWAS studies were included. For the second stage (replication), follow-up was pursued for only 96 SNPs. Since the genome-wide summary statistics are confined to the discovery setting, only 40 SNPs are classified as associated at GWS level, with the rest achieving genome-wide significance in the meta-analysis of both stages.

### Estimation of the Genetic Ancestry Association with Pediatric BMD

The effect of genetic ancestry on BMD was first evaluated by least-squares means using the R package lsmeans (http://cran.r-project.org/web/packages/lsmeans/index.html, last accessed April 10, 2014). BMD was adjusted for age, gender, height, fat, and lean mass index (which mimic the equation of BMI by dividing the referred mass by the squared height) for all participants independently in both pediatric studies before comparison. Moreover, in a subsample of the Generation R Study (*n* = 1,750), a model that additionally included birth weight, hours of physical activity of the children at age 5, age and height of the mother right before pregnancy, and smoking during pregnancy and breastfeeding was assessed. Furthermore, we evaluated the effect of Sub Saharan African and East Asian genetic ancestry proportions on adjusted BMD. For each individual genetically identified as European in the Generation R Study, the percentage of genetic ancestry estimated by ADMIXTURE (Alexander et al. 2009) for either Sub-Saharan African or East Asian genetic ancestry was included as variable in the basic model.

### Estimation of the BMD-GS Association with Pediatric BMD

BMD adjusted variability explained by the GS was evaluated based on the linear regression coefficient of determination for both multiethnic populations, namely the Generation R and the BMDCS, for the whole populations and by ethnic group. Analyses were further adjusted for ten genomic principal components, when specified. Specific sets of principal components were generated in each of the two pediatric studies for the analyses within each of the ethnic clusters. Then results were pooled together. To quantify the magnitude of BMD increase across the genetic score in each study, we divided the obtained BMD-GS in quintiles. In each score-bin, we calculated the mean adjusted BMD. Comparisons among quintiles were made with reference to the middle quintile using *t*-tests, and *P* values were corrected for multiple testing. Deviation from the observed ethnic distribution across quintiles was evaluated using the chi-squared distribution, comparing the observed ethnic distribution with the one we would expect if the BMD-GS would be randomly distributed.

### Worldwide Geographic Distribution of the BMD-Increasing Alleles

The BMD-GS was calculated for each individual across the 53 HGDP populations and box plots per population were generated. To visualize the spatial distribution of the GS alleles, we created a density map with the median BMD-GS value observed at each population using MapViewer 7.1.1767. Point interpolation of the locations where no data was available was performed by means of inverse to distance.

### Signatures of Natural Selection in the 61 BMD SNPs and Associated Regions

In order to test whether the observed BMD-GS score differentiation pattern among populations was explained by genetic drift, we performed six different analyses:

1) Detection of enrichment of ancestral alleles in the set of BMD increasing alleles

Estimates of ancestral state were determined from the dbSNP database using BioMart (Kasprzyk 2011). Assessment of ancestral alleles enrichment in the BMD-GS was performed comparing the actual distribution of ancestral status to a Bernoulli distribution expected under random events. We based this calculation on 55 SNPs only, whose alleles were not palindromic (A/T or G/C) in order to avoid strand bias.

2) Randomization of the BMD increasing allele and quantification of the differentiation between continents by means of a two-way nested ANOVA design.

Each individual of the HGDP panel was hierarchically classified according to its sampling population, and the populations classified either to the Sub-Saharan African or non-Sub-Saharan African category. The amount of BMD-GS variation between the two ethnic categories was computed by means of a two-way nested ANOVA comprising three levels including 1) categories: Sub-Saharan and non-Sub-Saharan categories; 2) populations within categories; and 3) individuals within populations. In order to test whether the observed percentage of explained variation between these two categories was due to genetic drift, 100,000 permuted scores were generated, in which the sign of the effect for each of the 61 SNPs was randomly assigned to one of the two alleles. For each permuted data set, we constructed a new individual GS and determined the GS difference between African and non-African groups. A one-tail *P* value was estimated as the number of times that the explained variance (SS_among_/SS_total_) of the sampled set was greater than the one obtained for the original BMD-GS (variance: 0.225).

3) Population departure of the expected genomic value of BMD-GS scores estimated by means of a two-way nested ANOVA design

We applied an empirical genomic-based approach in order to obtain the expected null genomic distribution of the BMD-GS differentiation among the Sub-Saharan and non-Sub-Saharan populations under the assumption of genetic drift. Under this contention we randomly sampled 100,000 data sets of 61 SNPs across the genome showing similar genomic characteristics as the BMD-GS SNPs. Following the SNP matching framework described by Berg and Coop (2014), each BMD-GS SNP was matched by the imputed status at HGDP-CEPH data set, the *B* value (McVicker et al. 2009) and the allelic frequency of each of the BMD-increasing alleles in a proxy population (Orcadians) to the one where the SNPs were initially associated to the BMD phenotype (Europeans). A genomic SNP was considered as putative proxy for a given BMD-SNP if it was at least greater than 100 kb far away from the physical position of the BMD-SNP, the *B* statistic difference was smaller than 0.05 (calculated by http://www.phrap.org/othersoftware.html, last accessed February 27, 2015) and had a similar allelic frequency (<0.05) of the ancestral allele in the Orcadian population. At each iteration, 61 SNPs were sampled at random from the pool of proxy SNPs and a new genetic score was computed for each individual. A two-way nested ANOVA framework (described above) was used to compare the differentiation between Sub-Saharan and non-Sub-Saharan categories. One tail *P* value was computed comparing the number of times that the explained variance (SS_among_/SS_total_) of the sampled set was bigger than the explained variance of the original BMD-GS.

4) Q_X_ statistic calculation

The Q_X_ statistic proposed by Berg and Coop (2014), represents a measure of the among population variance in estimated genetic values that is not explained by drift and shared history. We assessed the covariance matrix matching by SNP ascertainment characteristics including imputed status, *B*-value, and Orcadian allele frequency, as described above. In order to construct the null covariance F matrix, we sampled a maximum of 200 proxy SNPs for each BMD-GS SNP at a physical distance of at least 100 kb to ensure pairwise independence (total number of SNPs = 11,655). We computed the covariance F matrix and Q_X_ statistic as described (Berg and Coop 2014), using formulas 17 and 10, respectively).

5) Detection of departure of neutrality in sequence-based neutrality tests

We used the 1000 Genomes project selection browser (Pybus et al. 2014) to detect signatures of positive selection in the 61 genomic regions harboring BMD-GS SNPs in the CEU, CHB, and YRI populations. For each population, nine different summary statistics incorporated in the browser were used to evaluate the 61 regions simultaneously partitioning across windows of an average size of 30 kb: CLR, XPCLR of PopA versus PopB and XPCLR of PopA versus PopC, Fay and Wu’s *H*, Fu and Li’s *D*, Fu and Li’s *F*, R2, Tajima’s *D*, and Wall’s *B*. Hierarchical Bayesian binomial model using a logit transformation, as implemented in Winbugs (Lunn et al. 2000), was used to estimate the distribution of the frequency of statistically significant neutrality tests at 5% and asses if the 95% credible interval included the expected 5% by chance.

6) Mean MAF test

We compared the mean MAF in each population to that under the expectation of random genetic drift. In order to avoid the effect of GWAS ascertainment bias in our results, we first sampled 100,000 sets of 61 SNPs across the genome, assuring that the selected markers: 1) have been associated in Europeans by GWAS to at least one of the phenotypes/traits described in the GWAS catalog (Welter, et al. 2014; https://www.genome.gov/26525384, last accessed March 20, 2015) and 2) are also present in the HapMap population of interest. In the case of CEU individuals, the number of BMD-GS SNPs present in the HapMap Phase II data set was 61 and the number of GWAS SNPs from where to sample was 8,349. In the case of YRI individuals, the number of GS-BMD SNPs was 58 and the number of GWAS SNPs from where to sample was 7,500; for JPT + CHB individuals, there were 58 GS-BMD SNPs and 7,552 GWAS SNPs from where to sample. A one-tailed *P* value was computed counting the number of times that the sampled mean was higher than the observed one.

## Supplementary Material

Supplementary figures S1 and S2 and tables S1––S5 are available at *Molecular Biology and Evolution* online (http://www.mbe.oxfordjournals.org/).

Supplementary Data
