# Supplementary material for: BMD Loci Contribute to Ethnic and Developmental Differences in Skeletal Fragility across Populations: Assessment of Evolutionary Selection Pressures
Source: Mol Biol Evol. 2015 Jul 29;32(11):2961–72. doi: 10.1093/molbev/msv170 (PMC4651235; doi:10.1093/molbev/msv170)
Supplement: Supplementary Data [file supp_32_11_2961__index.html]

BMD Loci Contribute to Ethnic And Developmental Differences in Skeletal Fragility Across Populations: Assessment of Evolutionary Selection Pressures — BMD Loci Contribute to Ethnic and Developmental Differences in Skeletal Fragility across Populations: Assessment of Evolutionary Selection Pressures — BMD Loci Contribute to Ethnic and Developmental Differences in Skeletal Fragility across Populations: Assessment of Evolutionary Selection Pressures — Supplementary Data 

# BMD Loci Contribute to Ethnic and Developmental Differences in Skeletal Fragility across Populations: Assessment of Evolutionary Selection Pressures

## Supplementary Data

files

- Supplementary Data - pdf file
