# Supplementary material for: BMD Loci Contribute to Ethnic and Developmental Differences in Skeletal Fragility across Populations: Assessment of Evolutionary Selection Pressures
Source: Mol Biol Evol. 2015 Jul 29;32(11):2961–72. doi: 10.1093/molbev/msv170 (PMC4651235; doi:10.1093/molbev/msv170)
Supplement: Supplementary Data [file supp_msv170_SI_Appendix.pdf]

Supplementary Material

BMD Loci Subject To Selection Pressures Contribute To Ethnic And Developmental Differences in Skeletal Fragility Across Populations

Medina-Gomez et al.

Table S1. Description of the 63 GS-BMD SNPs of the BMD genetic score, including rs SNP id, locus, closest gene annotation, BMD increasing allele (IA) on the positive strand and allele frequencies in the HapMap Phase II YRI and CEU panels.

SNP	Locus	Closest Gene	IA	Freq YRI	Freq CEU
rs17482952	1p31.3	GPR177	A	0.929	0.915
rs12407028	1p31.3	GPR177	T	0.825	0.533
rs7521902	1p36.12	WNT4	C	0.876	0.765
rs6426749	1p36.12	ZBTB40	C	0.317	0.125
rs479336	1q24.3	DNM3	G	0.673	0.146
rs4233949	2p16.2	SPTBN1	C	0.332	0.406
rs7584262	2p21	LOC91461	T	0.363	0.235
rs17040773	2q13	ANAPC1	A	0.973	0.743
rs1878526	2q14.2	INSIG2	A	0.254	0.181
rs1346004	2q.243	GALNT3	G	0.85	0.542
rs430727	3p22.1	CTNNB1	C	0.593	0.568
rs1026364	3q13.2	KIAA2018	T	0.412	0.296
rs344081	3q25.31	LEKR1	T	0.35	0.872
rs3755955	4p16.3	IDUA	G	0.889	0.858
rs6532023	4q22.1	MEPE	T	0.322	0.392
rs1366594	5q14.3	MEF2C	A	0.084	0.522
rs11755164	6p21.1	SUPT3H/RUNX2	C	0.442	0.597
rs9466056	6p22.3	CDKAL1/SOX4	G	0.621	0.59
rs13204965	6q22.32	RSPO3	A	0.977	0.735
rs4869742	6q25.1	C6orf97	C	0.198	0.664
rs7751941	6q25.1	C6orf97	G	0.863	0.812
rs10226308	7p14.1	TXNDC3	G	0.093	0.192
rs6959212	7p14.1	STARD3NL	C	0.673	0.674
rs4727338	7q21.3	SLC25A13	C	0.858	0.729
rs13245690	7q31.31	FLJ21986	A	0.806	0.616
rs3801387	7q31.31	WNT16	G	0.358	0.261
rs7812088	7q36.1	ABCF2	A	0.018	0.119
rs7017914	*8q13.3*	*XKR9*	*A*	*0.475*	*0.82**
rs2062377	8q24.12	TNFRSF11B	T	0.067	0.466
rs7851693	*9q34.11*	*FUBP3*	*C*	*0.67*	*0.71**
rs3905706	10p11.23	MPP7	T	0.661	0.167
rs1373004	10q21.1	MBL2/DKK1	G	0.415	0.883
rs7071206	10q22.3_1	KCNMA1	C	0.097	0.217
rs7084921	10q24.2	CPN1	T	0.544	0.398
rs7932354	11p11.2	ARHGAP1	T	0.9	0.183
rs10835187	11p14.1_1	LIN7C	C	0.934	0.447

rs163879	11p14.1_2	DCDC5	C	0.442	0.31
rs7108738	11p15.2	SOX6	G	0.345	0.186
rs3736228	11q13.2	LRP5	C	1	0.86
rs7953528	12p11.22	KLHDC5/PTHLH	A	0	0.2
rs2887571	12p13.33	ERC1/WNT5B	G	0.177	0.217
rs12821008	12q13.12	DHH	T	0.036	0.367
rs2016266	12q13.13	SP7	G	0.642	0.332
rs736825	12q13.13	HOXC6	C	0.771	0.653
rs1053051	12q23.3	C12orf23	C	0.854	0.54
rs9533090	13q14.11	AKAP11	C	0.817	0.567
rs1286083	14q32.12	RPS6KA5	C	0.28	0.216
rs11623869	14q32.32	MARK3	G	0.81	0.637
rs4985155	16p13.11	NTAN1	G	0.214	0.317
rs9921222	16p13.3_1	AXIN1	C	0.305	0.575
rs13336428	16p13.3_2	C16orf38/CLCN7	G	0.19	0.553
rs1564981	16q12.1	CYLD	G	0.04	0.531
rs1566045	16q12.1	SALL1/CYLD	C	0.022	0.235
rs10048146	16q24.1	FOXL1	A	0.832	0.81
rs4790881	17p13.3	SMG6	A	0.888	0.623
rs4792909	17q21.31_1	SOST	T	0.887	0.385
rs227584	17q21.31_1	C17orf53	C	0.96	0.341
rs1864325	17q21.31_2	MAPT	C	0.996	0.792
rs7217932	17q24.3	SOX9	A	0.763	0.466
rs4796995	18p11.21	C18orf19	A	0.942	0.655
rs884205	18q21.33	TNFRSF11A	C	0.956	0.719
rs10416218	19q13.11	GPATCH1	C	0.858	0.183
rs3790160	20p12.2	JAG1	T	0.421	0.544

*Frequencies as reported by 1000 Genomes project. In blue italics the 2 SNPs not present in the imputation panels of any of the study populations.

Table S2. BMD variance explained by the BMD-GS in the pediatric cohorts overall and by defined ancestry group

**Generation R Study (The Netherlands)**							
		**score61noPCS**			**score61PCs**		
	n	beta	P	R^2	beta	P	R^2
**All**	3,994	5.00	$<2.2x10^{-16}$	0.045	3.74	$<2.2x10^{-16}$	0.026
**European**	3,499	4.76	$<2.2x10^{-16}$	0.039	4.39	$<2.2x10^{-16}$	0.033
**E. Asian**	159	-1.24	0.527	0.003	0.76	0.697	0.001
**S.S. African**	336	2.60	0.061	0.011	1.58	0.256	0.004

**BMD-Childhood Study (U.S.A)**							
		**score61noPCS**			**score61PCs**		
	n	beta	P	R^2	beta	P	R^2
**All**	1,670	5.45	$< 2.2x10^{-16}$	0.057	3.39	$9.8x10^{-10}$	0.022
**European**	1,277	4.63	$3.9x10^{-11}$	0.034	4.61	$4.86x10^{-11}$	0.033
**E. Asian**	78	2.73	0.373	0.010	2.22	0.470	0.007
**S.S. African**	315	3.81	0.009	0.022	3.34	0.022	0.017

E. Asian: East Asian, S.S.African: Sub-Saharan African

Table S3. Human Genome Diversity Panel Populations with mean BMD-GS values and latitude (n=940).

IID	Population	Country	GS	latitude
HGDP00993	BantuSouthEast	SouthernAfrica	0.57	-28.4
HGDP00994	BantuSouthEast	SouthernAfrica	0.57	-28.4
HGDP01030	BantuSouthEast	SouthernAfrica	0.58	-28.4
HGDP01033	BantuSouthEast	SouthernAfrica	0.61	-28.4
HGDP01034	BantuSouthEast	SouthernAfrica	0.57	-28.4
HGDP01028	BantuSouthWest	SouthernAfrica	0.53	-21
HGDP01031	BantuSouthWest	SouthernAfrica	0.60	-21
HGDP01035	BantuSouthWest	SouthernAfrica	0.61	-21
HGDP00991	San	Namibia	0.57	-21
HGDP00992	San	Namibia	0.57	-21
HGDP01029	San	Namibia	0.61	-21
HGDP01032	San	Namibia	0.58	-21
HGDP01036	San	Namibia	0.55	-21
HGDP00832	Surui	Brazil	0.55	-11
HGDP00837	Surui	Brazil	0.46	-11
HGDP00838	Surui	Brazil	0.48	-11
HGDP00843	Surui	Brazil	0.55	-11
HGDP00845	Surui	Brazil	0.47	-11
HGDP00846	Surui	Brazil	0.54	-11
HGDP00849	Surui	Brazil	0.53	-11
HGDP00852	Surui	Brazil	0.51	-11
HGDP00995	Karitiana	Brazil	0.48	-10
HGDP00998	Karitiana	Brazil	0.57	-10
HGDP00999	Karitiana	Brazil	0.54	-10
HGDP01001	Karitiana	Brazil	0.50	-10
HGDP01003	Karitiana	Brazil	0.52	-10
HGDP01006	Karitiana	Brazil	0.52	-10
HGDP01009	Karitiana	Brazil	0.54	-10
HGDP01010	Karitiana	Brazil	0.49	-10
HGDP01012	Karitiana	Brazil	0.52	-10
HGDP01013	Karitiana	Brazil	0.58	-10
HGDP01014	Karitiana	Brazil	0.52	-10
HGDP01015	Karitiana	Brazil	0.54	-10
HGDP01018	Karitiana	Brazil	0.58	-10
HGDP01019	Karitiana	Brazil	0.56	-10
HGDP00491	Melanesian	Salomon Islands	0.41	-6
HGDP00656	Melanesian	Salomon Islands	0.43	-6
HGDP00661	Melanesian	Salomon Islands	0.52	-6
HGDP00662	Melanesian	Salomon Islands	0.48	-6

HGDP00663	Melanesian	Salomon Islands	0.46	-6
HGDP00664	Melanesian	Salomon Islands	0.46	-6
HGDP00787	Melanesian	Salomon Islands	0.40	-6
HGDP00788	Melanesian	Salomon Islands	0.48	-6
HGDP01027	Melanesian	Salomon Islands	0.45	-6
HGDP00540	Papuan	NewPapuaGuinea	0.50	-4
HGDP00541	Papuan	NewPapuaGuinea	0.48	-4
HGDP00542	Papuan	NewPapuaGuinea	0.46	-4
HGDP00543	Papuan	NewPapuaGuinea	0.47	-4
HGDP00544	Papuan	NewPapuaGuinea	0.40	-4
HGDP00545	Papuan	NewPapuaGuinea	0.44	-4
HGDP00546	Papuan	NewPapuaGuinea	0.49	-4
HGDP00547	Papuan	NewPapuaGuinea	0.48	-4
HGDP00548	Papuan	NewPapuaGuinea	0.47	-4
HGDP00549	Papuan	NewPapuaGuinea	0.48	-4
HGDP00550	Papuan	NewPapuaGuinea	0.47	-4
HGDP00551	Papuan	NewPapuaGuinea	0.57	-4
HGDP00552	Papuan	NewPapuaGuinea	0.43	-4
HGDP00553	Papuan	NewPapuaGuinea	0.58	-4
HGDP00554	Papuan	NewPapuaGuinea	0.52	-4
HGDP00555	Papuan	NewPapuaGuinea	0.48	-4
HGDP00556	Papuan	NewPapuaGuinea	0.46	-4
HGDP01405	BantuKenya	Kenya	0.62	-3
HGDP01406	BantuKenya	Kenya	0.50	-3
HGDP01408	BantuKenya	Kenya	0.58	-3
HGDP01411	BantuKenya	Kenya	0.53	-3
HGDP01412	BantuKenya	Kenya	0.57	-3
HGDP01414	BantuKenya	Kenya	0.54	-3
HGDP01415	BantuKenya	Kenya	0.57	-3
HGDP01416	BantuKenya	Kenya	0.57	-3
HGDP01417	BantuKenya	Kenya	0.59	-3
HGDP01418	BantuKenya	Kenya	0.53	-3
HGDP01419	BantuKenya	Kenya	0.59	-3
HGDP00449	MbutiPygmy	Congo	0.56	1
HGDP00450	MbutiPygmy	Congo	0.52	1
HGDP00456	MbutiPygmy	Congo	0.61	1
HGDP00462	MbutiPygmy	Congo	0.55	1
HGDP00467	MbutiPygmy	Congo	0.52	1
HGDP00471	MbutiPygmy	Congo	0.53	1
HGDP00474	MbutiPygmy	Congo	0.52	1
HGDP00476	MbutiPygmy	Congo	0.56	1
HGDP00478	MbutiPygmy	Congo	0.52	1
HGDP00982	MbutiPygmy	Congo	0.57	1

HGDP00984	MbutiPygmy	Congo	0.52	1
HGDP01081	MbutiPygmy	Congo	0.52	1
HGDP00702	Colombian	Colombia	0.53	3
HGDP00703	Colombian	Colombia	0.52	3
HGDP00704	Colombian	Colombia	0.52	3
HGDP00706	Colombian	Colombia	0.55	3
HGDP00708	Colombian	Colombia	0.56	3
HGDP00710	Colombian	Colombia	0.55	3
HGDP00970	Colombian	Colombia	0.57	3
HGDP00454	BiakaPygmy	CentralAfricanRepublic	0.55	4
HGDP00455	BiakaPygmy	CentralAfricanRepublic	0.64	4
HGDP00457	BiakaPygmy	CentralAfricanRepublic	0.63	4
HGDP00458	BiakaPygmy	CentralAfricanRepublic	0.55	4
HGDP00459	BiakaPygmy	CentralAfricanRepublic	0.61	4
HGDP00460	BiakaPygmy	CentralAfricanRepublic	0.60	4
HGDP00461	BiakaPygmy	CentralAfricanRepublic	0.59	4
HGDP00464	BiakaPygmy	CentralAfricanRepublic	0.54	4
HGDP00465	BiakaPygmy	CentralAfricanRepublic	0.56	4
HGDP00466	BiakaPygmy	CentralAfricanRepublic	0.57	4
HGDP00469	BiakaPygmy	CentralAfricanRepublic	0.55	4
HGDP00470	BiakaPygmy	CentralAfricanRepublic	0.57	4
HGDP00472	BiakaPygmy	CentralAfricanRepublic	0.54	4
HGDP00473	BiakaPygmy	CentralAfricanRepublic	0.60	4
HGDP00475	BiakaPygmy	CentralAfricanRepublic	0.56	4
HGDP00479	BiakaPygmy	CentralAfricanRepublic	0.66	4
HGDP00985	BiakaPygmy	CentralAfricanRepublic	0.58	4
HGDP00986	BiakaPygmy	CentralAfricanRepublic	0.60	4
HGDP01086	BiakaPygmy	CentralAfricanRepublic	0.57	4
HGDP01090	BiakaPygmy	CentralAfricanRepublic	0.54	4
HGDP01094	BiakaPygmy	CentralAfricanRepublic	0.55	4
HGDP00920	Yoruba	Nigeria	0.61	8
HGDP00924	Yoruba	Nigeria	0.54	8
HGDP00925	Yoruba	Nigeria	0.48	8
HGDP00926	Yoruba	Nigeria	0.58	8
HGDP00927	Yoruba	Nigeria	0.61	8
HGDP00928	Yoruba	Nigeria	0.58	8
HGDP00929	Yoruba	Nigeria	0.57	8
HGDP00930	Yoruba	Nigeria	0.54	8
HGDP00931	Yoruba	Nigeria	0.58	8
HGDP00932	Yoruba	Nigeria	0.57	8
HGDP00933	Yoruba	Nigeria	0.52	8
HGDP00934	Yoruba	Nigeria	0.59	8
HGDP00935	Yoruba	Nigeria	0.58	8

HGDP00936	Yoruba	Nigeria	0.61	8
HGDP00937	Yoruba	Nigeria	0.57	8
HGDP00938	Yoruba	Nigeria	0.58	8
HGDP00939	Yoruba	Nigeria	0.55	8
HGDP00940	Yoruba	Nigeria	0.55	8
HGDP00941	Yoruba	Nigeria	0.59	8
HGDP00942	Yoruba	Nigeria	0.48	8
HGDP00943	Yoruba	Nigeria	0.53	8
HGDP00711	Cambodian	Cambodia	0.52	12
HGDP00712	Cambodian	Cambodia	0.48	12
HGDP00713	Cambodian	Cambodia	0.43	12
HGDP00714	Cambodian	Cambodia	0.47	12
HGDP00715	Cambodian	Cambodia	0.44	12
HGDP00716	Cambodian	Cambodia	0.52	12
HGDP00717	Cambodian	Cambodia	0.44	12
HGDP00719	Cambodian	Cambodia	0.51	12
HGDP00720	Cambodian	Cambodia	0.52	12
HGDP00721	Cambodian	Cambodia	0.47	12
HGDP00904	Mandenka	Senegal	0.53	12
HGDP00905	Mandenka	Senegal	0.60	12
HGDP00906	Mandenka	Senegal	0.55	12
HGDP00907	Mandenka	Senegal	0.54	12
HGDP00908	Mandenka	Senegal	0.55	12
HGDP00909	Mandenka	Senegal	0.57	12
HGDP00910	Mandenka	Senegal	0.61	12
HGDP00911	Mandenka	Senegal	0.52	12
HGDP00912	Mandenka	Senegal	0.57	12
HGDP00913	Mandenka	Senegal	0.56	12
HGDP00914	Mandenka	Senegal	0.52	12
HGDP00915	Mandenka	Senegal	0.55	12
HGDP00917	Mandenka	Senegal	0.50	12
HGDP00918	Mandenka	Senegal	0.60	12
HGDP01199	Mandenka	Senegal	0.50	12
HGDP01200	Mandenka	Senegal	0.57	12
HGDP01201	Mandenka	Senegal	0.52	12
HGDP01202	Mandenka	Senegal	0.56	12
HGDP01283	Mandenka	Senegal	0.53	12
HGDP01284	Mandenka	Senegal	0.58	12
HGDP01285	Mandenka	Senegal	0.53	12
HGDP01286	Mandenka	Senegal	0.55	12
HGDP00854	Maya	Mexico	0.57	19
HGDP00855	Maya	Mexico	0.53	19
HGDP00856	Maya	Mexico	0.53	19

HGDP00857	Maya	Mexico	0.49	19
HGDP00858	Maya	Mexico	0.53	19
HGDP00859	Maya	Mexico	0.57	19
HGDP00860	Maya	Mexico	0.49	19
HGDP00861	Maya	Mexico	0.54	19
HGDP00862	Maya	Mexico	0.54	19
HGDP00863	Maya	Mexico	0.50	19
HGDP00864	Maya	Mexico	0.53	19
HGDP00865	Maya	Mexico	0.46	19
HGDP00868	Maya	Mexico	0.52	19
HGDP00869	Maya	Mexico	0.54	19
HGDP00870	Maya	Mexico	0.49	19
HGDP00871	Maya	Mexico	0.46	19
HGDP00872	Maya	Mexico	0.52	19
HGDP00873	Maya	Mexico	0.46	19
HGDP00875	Maya	Mexico	0.50	19
HGDP00876	Maya	Mexico	0.55	19
HGDP00877	Maya	Mexico	0.50	19
HGDP01307	Dai	China	0.44	21
HGDP01308	Dai	China	0.49	21
HGDP01309	Dai	China	0.48	21
HGDP01310	Dai	China	0.49	21
HGDP01311	Dai	China	0.48	21
HGDP01312	Dai	China	0.46	21
HGDP01313	Dai	China	0.54	21
HGDP01314	Dai	China	0.46	21
HGDP01315	Dai	China	0.49	21
HGDP01316	Dai	China	0.52	21
HGDP01317	Lahu	China	0.54	22
HGDP01318	Lahu	China	0.45	22
HGDP01319	Lahu	China	0.44	22
HGDP01320	Lahu	China	0.44	22
HGDP01321	Lahu	China	0.50	22
HGDP01322	Lahu	China	0.42	22
HGDP01323	Lahu	China	0.48	22
HGDP01326	Lahu	China	0.54	22
HGDP00163	Sindhi	Pakistan	0.49	25.5
HGDP00165	Sindhi	Pakistan	0.50	25.5
HGDP00167	Sindhi	Pakistan	0.43	25.5

Table S4. Neutrality tests across the different regions of the SNPs included in the BMD-GS.

Population	Test*	P <0.05	No. tests	Frequency
CEU	XPCLR-CHB	3	60	0.050
CEU	XPCLR-YRI	3	60	0.050
CEU	CLR	0	61	0.000
CEU	Fay and Wu's H	3	61	0.049
CEU	Fu and Li's D	2	61	0.033
CEU	Fu and Li's F	0	61	0.000
CEU	R^2	0	61	0.000
CEU	Tajima's D	0	61	0.000
CEU	Wall's B	2	61	0.033
CHB/JPT	XPCLR-CEU	1	60	0.017
CHB/JPT	XPCLR-YRI	0	60	0.000
CHB/JPT	CLR	1	61	0.016
CHB/JPT	Fay and Wu's H	0	61	0.000
CHB/JPT	Fu and Li's D	1	61	0.016
CHB/JPT	Fu and Li's F	1	61	0.016
CHB/JPT	R^2	1	61	0.016
CHB/JPT	Tajima's D	1	61	0.016
CHB/JPT	Wall's B	4	61	0.066
YRI	XPCLR-CEU	2	60	0.033
YRI	XPCLR-YRI	2	60	0.033
YRI	CLR	1	61	0.016
YRI	Fay and Wu's H	4	61	0.066
YRI	Fu and Li's D	1	61	0.016
YRI	Fu and Li's F	1	61	0.016
YRI	R^2	4	61	0.066
YRI	Tajima's D	3	61	0.049
YRI	Wall's B	4	61	0.066

*Results from the 9 tests per population. Based on allele frequency spectrum: Tajima's D, CLR, Fay and Wu's H, Fu and Li's D, Fu and Li's F, R^2 . Based on linkage disequilibrium structure: Wall's B. Based on Population differentiation: XPCLR. Tests were applied to the three panels using the 1,000 Genomes Browser, considering only GS polymorphic SNPs for each population. Assessments were done across 30 Kb windows.

Table S5. Aggregate results using hierarchical modeling for the different HapMap Phase II populations.

Population	Mean	2.5%	97.5%
CEU	0.016	0.006	0.030
CHB/JPT	0.018	0.004	0.037
YRI	0.037	0.023	0.057

Mean and 95% Credible interval of the posterior distributions of the aggregated frequency of statistically significant tests obtained from Hierarchical Bayesian binomial modeling in each panel

A.

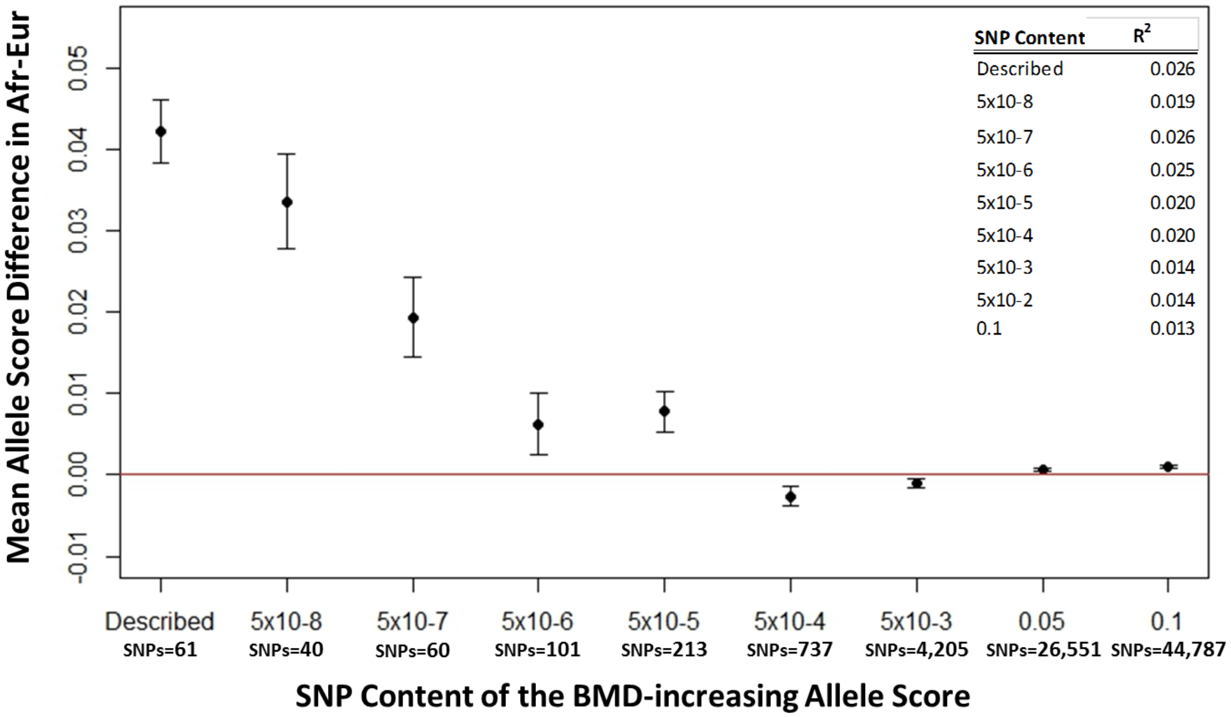

B.

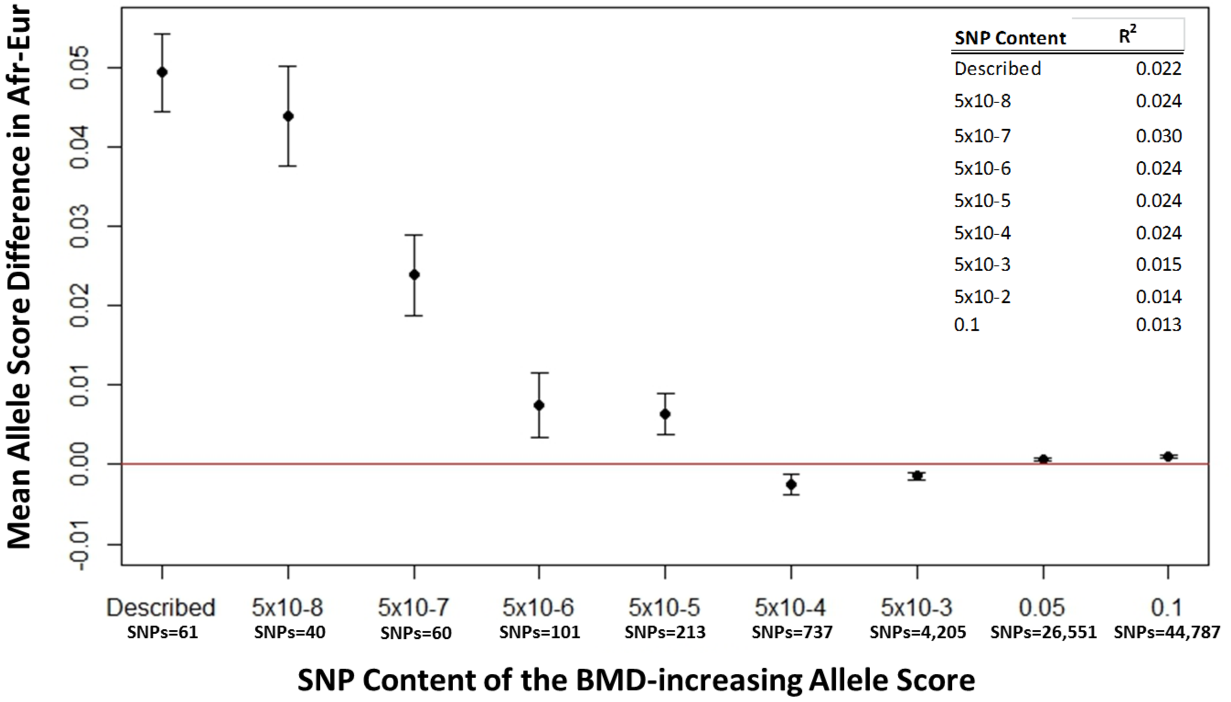

Figure S1 Mean difference in BMD-increasing genetic scores between African and European children Y-axis: Differences in mean score were calculated for genetic scores based on the Stage 1 GWAS meta-analysis results (Estrada et al. 2012). X-axis: Scores calculated including SNP content at different significance thresholds. Red line: expected mean difference (y=0). Afr: Children of Sub-Saharan African ancestry Eur: Children of European ancestry**. A. Generation R Study (The Netherlands). BMD Childhood Study (U.S.A.)**

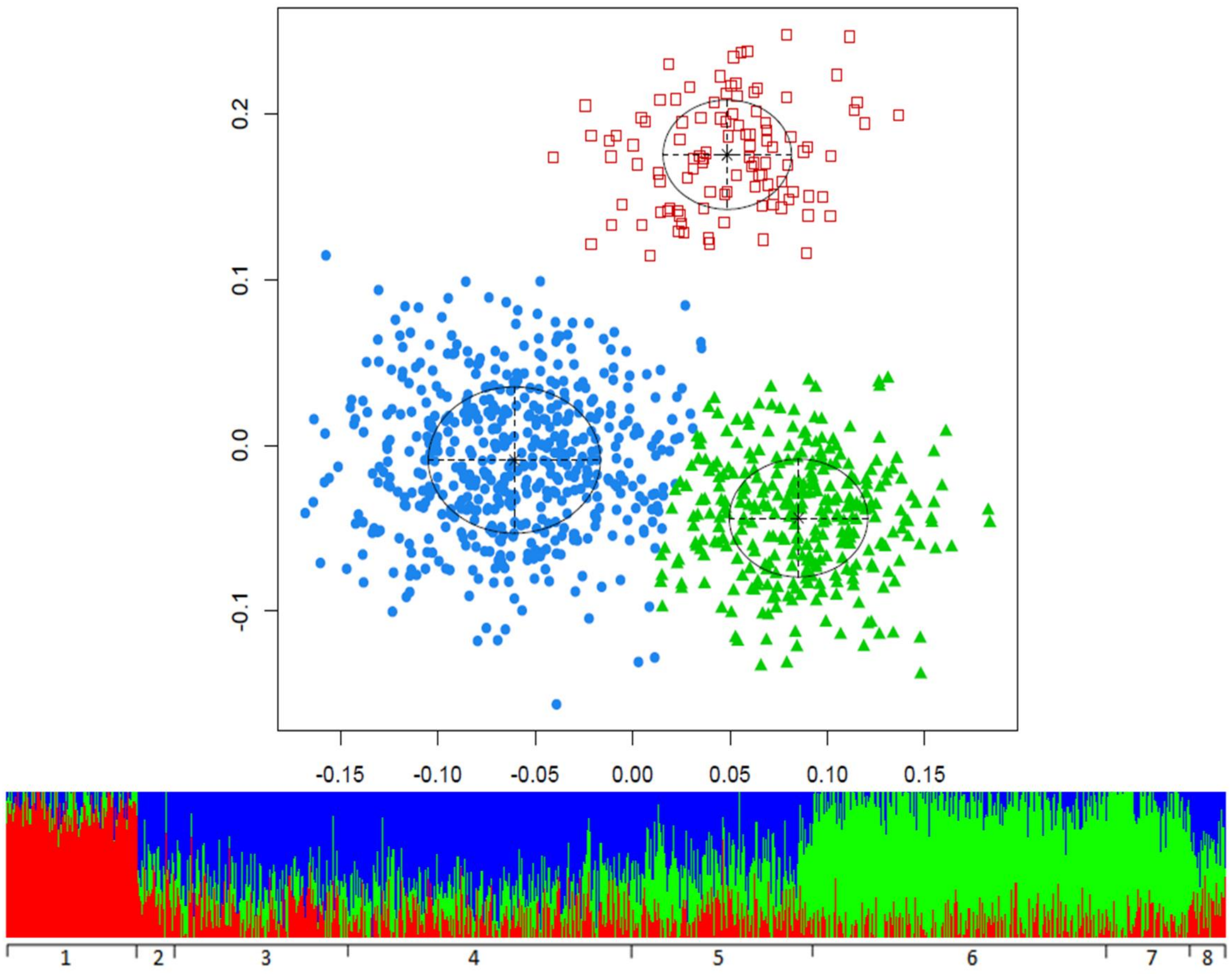

Figure S2. HGDP clustering based in 61 BMD-associated SNPs. A. First two dimensions (C 1 vs. C2) from a MDS using an individual pairwise IBS distance matrix. Blue represents Eurasian populations, green E. Asian, Native American and Oceanian populations and red S. African populations. Clusters were obtained after applying the *mclust* algorithm. **B. Admixture compound for the 940 HGDP-CEPH individuals**. Populations are grouped and labeled as: 1. Sub-saharan Africa (BantuSouthWest, BantuSouthEast, BantuKenya, BiakaPygmy, San, Yoruba, Mandenka, MbutiPygmy), 2. North Africa (Mozabite), 3. Middle East (Druze, Palestinian, Bedouin), 4. Europe (Italian, French, Orcadian, Adygei, Basque, Sardininan, Tuscan, Russian), 5. Central-South Asia (Brahui, Hazara, Uygur, Kalash, Balochi, Makrani, Sindhi, Pathan, Burusho), 6. East Asia (Dai, Daur, Han, Hezhen, Lahu, Miaozu, Mongola, Naxi, Orogen, She, Tu, Tujia, Xibo, Yizu, Japanese, Yakut), 7. America (Karitiana, Surui, Colombia, Pima), 8. Oceania (Melanesian, Papuan).

References

Estrada K, Styrkarsdottir U, Evangelou E, Hsu YH, Duncan EL, Ntzani EE, Oei L, Albagha OM, Amin N, Kemp JP, et al. 2012. Genome-wide meta-analysis identifies 56 bone mineral density loci and reveals 14 loci associated with risk of fracture. Nat Genet 44:491-501.
